# Impact of Environmental Microbes on the Composition of the Gut Microbiota of Adult BALB/c Mice

**DOI:** 10.1371/journal.pone.0160568

**Published:** 2016-08-12

**Authors:** Zhimao Bai, Honglin Zhang, Na Li, Zhiyu Bai, Liling Zhang, Zhencheng Xue, Haitao Jiang, Yuan Song, Dongrui Zhou

**Affiliations:** 1 Key Laboratory of Environmental Medicine Engineering of Ministry of Education, School of Public Health, Southeast University, Nanjing, Jiangsu, People’s Republic of China; 2 College of Food Science, Nanjing Xiaozhuang University, Nanjing, Jiangsu, People’s Republic of China; 3 Key Laboratory of Child Development and Learning Science, Southeast University, Nanjing, Jiangsu, People’s Republic of China; 4 Suzhou Research Institute of Southeast University, Suzhou, Jiangsu, People’s Republic of China; 5 Department of Respiratory Medicine, Affiliated Hospital, Chengde Medical College, Chengde, Hebei, People’s Republic of China; 6 Department of Child Health, Suzhou Municipal Hospital, Suzhou, Jiangsu, People’s Republic of China; Harvard Medical School, UNITED STATES

## Abstract

To investigate the impact of microbes within the living environment on the gut microbiota of adults, we raised three groups of BALB/c mice from 3–4 weeks age in the same specific-pathogen-free animal room for 8 weeks. The control group lived in cages with sterilized bedding (pelletized cardboard), the probiotics group had three probiotics added to the sterilized bedding, and the intestinal microbes (IM) group had the intestinal microbes of a healthy goat added to the bedding. All other variables such as diet, age, genetic background, physiological status, original gut microbiota, and living room were controlled. Using high-throughput sequencing of the 16S rRNA gene, we observed that the control and probiotics groups had similar diversity and richness of gut microbiota. The two groups had significantly lower diversity than the IM group. We also observed that the IM group had a specific structure of gut microbial community compared with the control and probiotics groups. However, the dominate bacteria changed slightly upon exposure to intestinal microbes, and the abundance of the non-dominate species changed significantly. In addition, exposure to intestinal microbes inhibited DNFB-induced elevation of serum IgE levels. Our results provide new evidence in support of the microflora and hygiene hypotheses.

## Introduction

The mammalian gastrointestinal tract harbors an astounding number and diversity of microbes that constitute the “microbiota.” The microbiota interacts with the host immune system, contributing to intestinal and systemic immune homeostasis[1,2]. Several reports have suggested that lower richness and diversity of gut microbiota is closely associated with immune system diseases [3,4]. This possibility could be associated with the notion that the induction of each lymphocyte subset may be regulated by a distinct component of the microbiota[5]. For instance, Clostridium clusters IV and XIVa promote accumulation of Treg cells in the colon[5], whereas segmented filamentous bacteria (SFBs) strongly induce intestinal T helper 17 (TH17) cells, which participate in host resistance to intestinal pathogens and promote systemic autoimmunity[6,7].

Recent reports proved diets influenced the diversity and composition of gut microbiota[8,9] since dietary fermentable fiber or fat content changed its composition, specifically by altering the ratio of Firmicutes to Bacteroidetes[8,10]. However, there have been very few reports concerning the impact of microbes within the living environment on the structure of gut microbiota in the past 10 years [11]. In the 1980s, it was found that the enterobacterial colonization in modern Sweden occurred at a later stage, compared to that in infants in developing countries[12,13]. Furthermore, in Swedish infants, after colonization with enterobacteria finally occurs, anaerobes may have already been established, in which case the enterobacteria cannot reach the high numbers often achieved in the absence of competition[14,15]. Therefore, the “microflora hypothesis” states that the overly hygienic Western lifestyle limits exposure to microbes and alters colonization of the infant’s gut, thus disrupting development of the immune system and ultimately leading to allergic diseases[16]. To assess this proposal, Zhou et al[11] raised three groups of mice under three different sanitary living environments with different bedding material, and all other variables such as diet, age, genetic background, physiological status, and original gut microbiota were controlled. Through high-throughput sequencing of the 16S rRNA gene, they found that each mouse group had a specific structure of the gut microbial community. Groups living in farmhouse and a general animal room harbored significantly more diverse and richer gut microbiota than those living in a specific pathogen-free (SPF) animal room. They also observed that the composition varied among the three groups, such as the ratio between the amount of Firmicutes and Bacteroides. On analyzing the elements in bedding material, which included microbes, dusts, decaying fallen leaves, hay, wheat straw, and top soil, it was proposed that microbes may play a key role in changing the composition of mouse gut microbiota. In addition, studies demonstrated that the bacterial colonization in the gut of infants is influenced by microbes within the living environment [16]. However, the impact of these microbes in adult mice has not been reported.

In this study, we mimicked living environmental microbes using the microorganisms of healthy goat gut, since there are few pathogen in healthy goat gut but there may be more in dust of living environment. We raised three groups of BALB/c mice aged 3–4 weeks in the same specific pathogen-free (SPF) animal room for 8 weeks. Each group of mice was exposed to different microbes, no added microbes, added probiotics or goat gut microbiota, in their cages in order to test the impact of microorganisms within the living environment on the composition of gut microbiota. For analysis, 16S rRNA gene sequencing by high throughput sequencing technology was performed.

## Materials and Methods

### Animals and groups

We purchased 6-week-old male and female BALB/c mice from B & K Universal (Shanghai, China) for breeding. One male mouse was mated with three females, and after the female mice were pregnant, two mice were moved to a separate cage. After birth, newborn female and male mice were weaned and moved to separate cages (at five animals per cage) at 3–4 weeks of age. Thirty mice (15 female and 15 male) were randomly distributed into three groups. Mice in the control group were reared in cages with sterile-grade murine bedding. A second group of mice (the probiotic group) was housed in cages using sterile-grade murine bedding with 10^6^ CFU of three probiotics, i.e., a type of human oral probiotic formulation containing *Bifidobacterium bifidum* (ATCC 29521), *Lactobacillus reuteri* (ATCC 55730), and *Enterococcus faecium* (CGMCC 2516). The last group of mice (the intestinal microbe [IM] group) was raised in cages using sterile-grade murine bedding with 10^6^ CFU of goat intestinal bacteria. The bedding was changed once per week. For the goat intestinal bacterial, the goat was raised in a farmhouse in a rural village and was fed grass and straw. In addition, the goat was given corn, wheat, and pulse each day but was not given any antibiotics or complete feed. The health of the goat was assessed before euthanasia. The separated luminal contents of the goat intestines were stored at –80°C. The intestinal microbes were isolated just before use by suspending the luminal contents from the intestine in a 10× physiological solution (0.9% w/v sodium chloride), which was then separated by high-speed centrifugation. The microbes were resuspended in physiological solution (0.9% w/v sodium chloride) at approximately 10^5^ CFU/mL. All animals were housed in the same specific pathogen-free animal room with a 12-h light/dark cycle at a temperature of 24°C ± 2°C and humidity of 40% ± 5% and were fed the same sterile-grade commercial pellet diet and distilled water ad libitum.

The experiments were carried out in strict accordance with the guidelines of the Animal Research Ethics Board of Southeast University. The Animal Care Research Advisory Committee of Southeast University and the National Institute of Biological Sciences approved all experiments involving mice (approval number: 2014041108). All surgeries were performed under sodium pentobarbital anesthesia, and all efforts were made to minimize suffering.

We collected fecal samples when the mice reached 11–12 weeks of age in order to analyze the gut microbial community.

### Allergen sensitization and challenge

After fecal samples were collected, allergic dermatitis was induced in the experimental mice by applying repeatedly 2-4-dinitrofluorobenzene (DNFB) to mouse skin, as previously described [17]. Briefly, we applied 25 μL of 0.15% DNFB in acetone/olive oil (3:1) to the ears (inner and outer surfaces) or 100 μL of the same solution to shaved back skin on days 1 and 4. We then further challenged the mice by dripping 0.2% DNFB onto the back and ear skin surfaces on days 7, 10, and 13. The severity of dermatitis on the rostral back skin and ears was assessed by evaluating dryness, crust, and keratinization. The total score for skin severity was defined as the sum of the individual scores (0 represents no symptoms and scores from 1 to 10 indicate the severity of the disease symptoms, with 10 being the most severe) for each of the following four signs and symptoms: erythema (hemorrhage), edema, excoriation (erosion), and scaling (dryness) on the ear and back regions [18]. The mice were euthanized 24 h after the final DNFB application.

### Care and use of animals

We monitored the health of the mice every other day, and performed body weight measurements every week. Mouse health was assessed by observing body weight changes, fecal shape changes, and external physical appearance. In this study, no animals became ill or died prior to the experimental endpoint. If any animals had become severely ill, we would have euthanized the animals to minimize pain and distress. We had a protocol for early euthanasia endpoints for such animals who had become severely ill during the experiments, based on the following criteria (animals had to exhibit one or more of these clinical signs: 1) weight loss of 15–20% within 1 or 2 weeks; 2) increased frequency of defecation, thinning, and diarrhea over 1 week; 3) symptoms of eating less, loss of hair quality, unresponsiveness, pain (arched back and curled up posture), fatigue, or listlessness for more than 1 week. The procedure for euthanasia in Balb/c mice was as follows. First, mice were anesthetized by administration of 1% sodium pentobarbital (w/v) by intraperitoneal injection using hypodermic needle at a dosage of 50 mg/kg. Blood samples were then collected from the eyeball after mice lost consciousness. Mice were then killed by neck dislocation after loss of consciousness, and death was confirmed by a qualified individuals. Additionally, as least two individuals were required to perform injections, i.e., one holding the animal and the other performing the procedure. Animals were not allowed to be left unattended between the time of initiation of euthanasia procedures and the time of death. The goat was anesthetized by administration of 1% sodium pentobarbital (w/v) by intraperitoneal injection with a hypodermic needle at a dosage of 30 mg/kg. The goat was then sacrificed by bleeding through the carotid artery immediately after loss of consciousness, and death was confirmed. Fecal samples were then collected. Euthanasia was performed by a licensed veterinarian for both mice and the goat.

### Serum IgE measurements

The total serum samples were prepared on day 14, about 24 h after the final DNFB application. The eBioscience Mouse IgE Ready-SET-GO mouse ELISA kit (Affymetrix) was used to analyze the concentration of total serum IgE according to the manufacturer’s instructions.

### DNA extraction

As genomic DNA samples, luminal contents of the goat intestines and fecal samples from mice (n = 8 per treatment group) were frozen in Eppendorf tubes and stored at –80°C until DNA extraction. The genomic DNA was extracted using a QIAamp Fast DNA Stool Mini Kit (Qiagen), according to the manufacturer’s instructions. The fecal sample suspension was heated at 95°C for 5 min, and DNA quality was assessed by gel electrophoresis and spectrophotometry (OD 260/280). Extracted DNA was stored at –20°C.

### MiSeq sequencing analysis of the community structure of gut microflora

The genomic DNA sequencing and data analysis were performed as previously reported by Zhou et al. [11]. In summary, the V4 hypervariable region of the 16S rRNA gene was amplified using the primer set reported by Angenent et al. [19], i.e., 515F (5′-GTGCCAGCMGCCGCGG) and 907R (5′- CCGTCAATTCMTTTRAGTTT), for each sample. The amplification was performed as follows: a typical 50-μL amplification reaction included 0.25 mM of each dNTP, 1× TransStart FastPfu buffer, 0.2 μM forward and 0.2 μM reverse primers, 1 μL TransStart FastPfu Taq DNA Polymerase (TransGen Biotech), and 300 ng template DNA. Polymerase chain reaction (PCR) was performed by initial denaturation at 94°C for 3 min; followed by 27 cycles of denaturation at 94°C for 45 s, annealing at 50°C for 45 s, and extension at 72°C for 45 s; and a final extension at 72°C for 5 min. The amplified DNA samples were purified and quantified using a QuantiFluor-ST Handheld Fluorometer with UV/Blue Channels (Promega Corporation). The PCR products were sequenced on an Illumina MiSeq platform using 2 × 250 bp chemistry. Raw paired-end reads were merged using FLASh software (http://ccb.jhu.edu/software/FLASH/). We eliminated low-quality sequences and removed PCR artifacts using Trimmomatic [20]. Chimeric sequences were removed using UCHIME [21]. We used the Quantitative Insights Into Microbial Ecology (QIIME) software package[22] to cluster the quality checked sequences into de novo operational taxonomic units (OTUs) at the 97% similarity threshold to generate OTUs and obtained the OTU abundance table for each sample. All samples were rarefied down to 49,128 sequences per sample to prevent bias due to sampling depth. Then, the rarefaction curves were constructed and α-diversity indices of the Chao estimator, Shannon index, and phylogenetic diversity (PD) index were calculated. Sequences longer than 200 bp were classified using the RDP classifier software at the 70% confidence threshold [23]. Principal coordinate analysis (PCoA) was performed using QIIME based on unweighted and weighted UniFrac distance metrics.

### Statistical analyses

All calculations such as analysis of variance (ANOVA) and linear correlation analysis were performed using the SPSS software, version 18.0.

### Tracing of bacteria in the intestinal bacterial communities of goats

After obtaining discriminatory level OTUs between the IM group and control or probiotic group, we traced the bacteria from the sequenced intestinal microbes of goats.

## Results

### 1. Exposure to goat intestinal microbes increased gut microbial richness in adult mice

**Fig 1:**
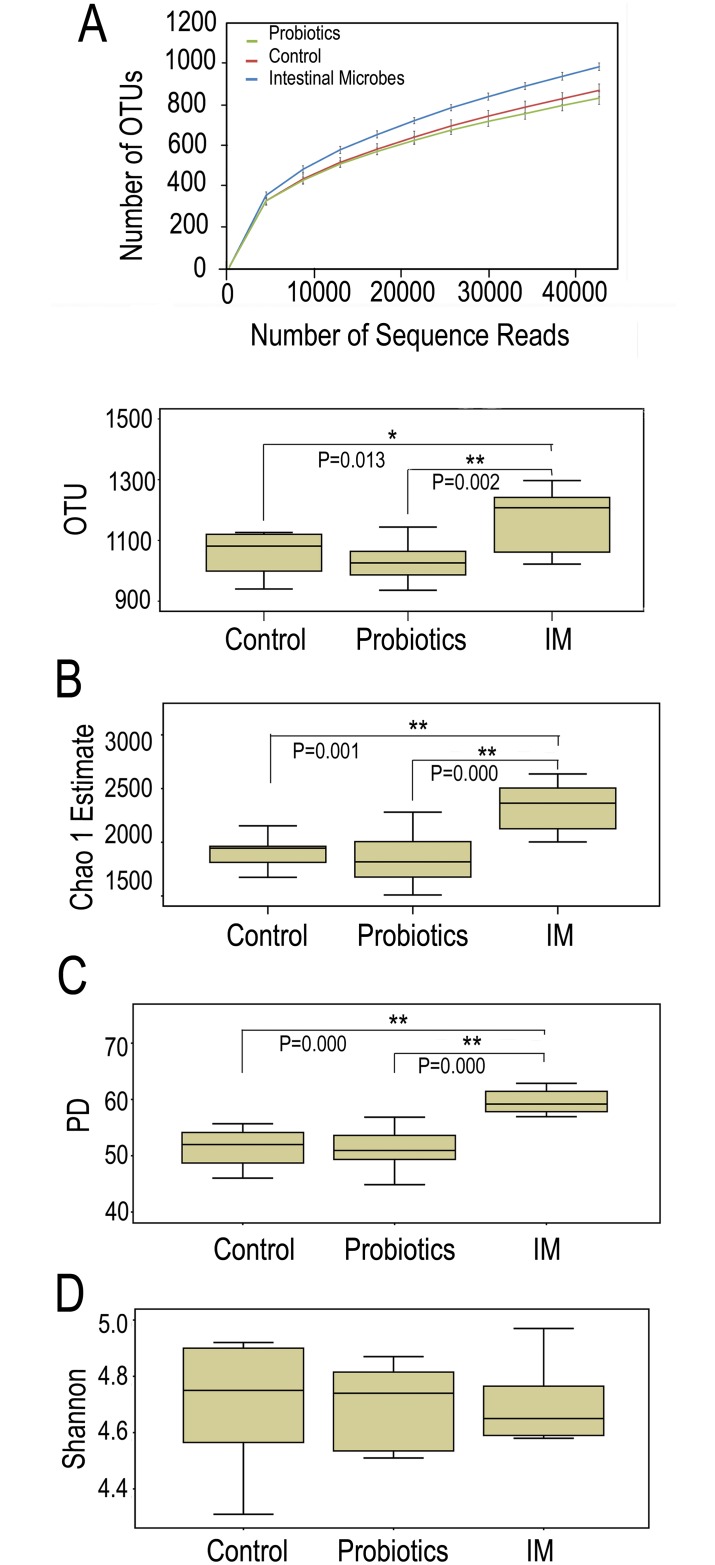
Comparison of bacterial richness and diversity among Control, Probiotics, and Microbes. Control mice were raised in cages with sterilized pelletized cardboard bedding, probiotics mice were raised in cages sterilized pelletized cardboard bedding added with probiotics, and intestinal microbes mice were raised in cages added with goat intestinal microbes. (A) Rarefaction curves showing unique operational taxonomic units (OTUs) (a box graph at the rarefied sequence number). (B) Chao1 estimators. (C) Phylogenetic diversity (PD). (D) Shannon index. (n = 8; * p < 0.05, ** p < 0.01, based on a two-tailed least significant difference test).

We applied Chao1 richness estimators to predict the richness of the gut microbiota of the three groups of mice. Our data indicated that mice in the IM group had higher bacterial richness than those in the control and probiotic groups. There were no significant differences between the control and probiotic groups. To estimate microbial diversity, we used the ecological indices of PD. The PD index incorporates the concept of phylogenetic distinctiveness and demonstrates sensitivity to species relatedness by summing the tree-branch length present in each subject of a phylogenetic dendrogram [24]. The PD index revealed that mice in the IM group harbored more diverse gut microbiota than mice in the control and probiotic groups (*p* < 0.01 by one-tailed Mann-Whitney tests; Fig 1C and Panel D in S2 Table). Similarly, there were no significant differences in microbial diversity between the control and probiotic groups. The ecological Shannon index, which combines both bacterial richness and evenness, was used to estimate microbial evenness between the experimental groups. The Shannon measurements demonstrated that there were no significant differences between experimental groups (Fig 1D and Panel D in S2 Table). Taken together, these results demonstrated that the control and probiotic groups had higher evenness than the IM group. The exposure to goat gut microbes was primarily linked to increased richness and diversity of the mouse gut microflora and was less correlated with evenness.

### 2. Exposure to goat intestinal microbes inhibited DNFB-induced elevation of total serum IgE levels

**Fig 2:**
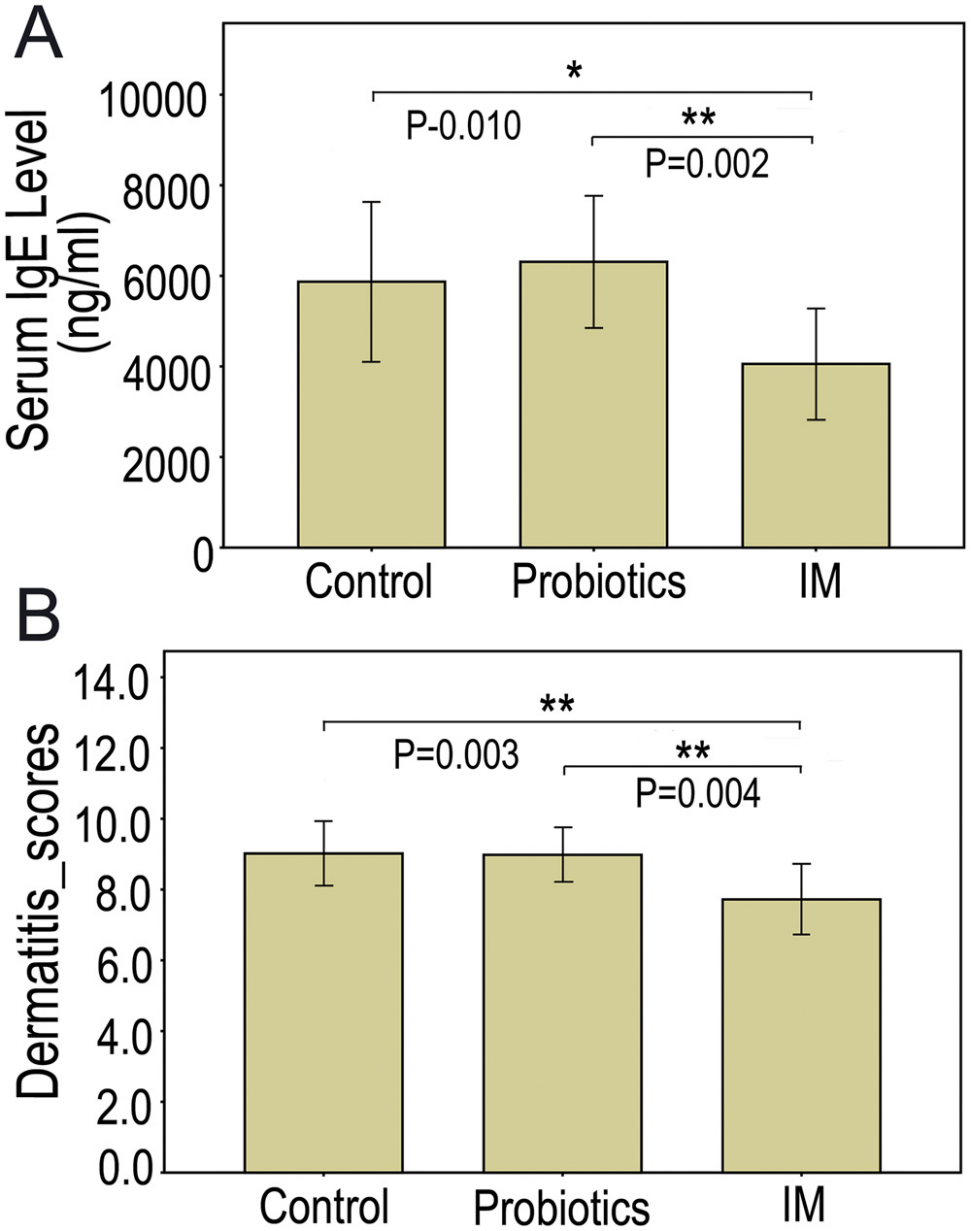
Effects of exposure to different concentrations of microbes. (A) Total serum IgE levels; (B) Dermatitis scores of rostral back and ear in BALB/c mice treated with DNFB. Serum absorbance in each group was averaged and each bar represents mean ± SEM (n = 10; *p < 0.05, **p < 0.01, based on a two-tailed least significant difference test).

To explore the exposure to living environmental microbes protects individuals from allergic disease, the severity of which are characterized by elevated total serum IgE levels [18,25], mice in each experimental group were challenged using DNFB after the mice were exposed in different microbes conditions. We collected the serum samples and the total IgE levels were measured with ELISA kit after the fifth DNFB treatment on day 13. The severity of dermatitis on the rostral back skin and ears was also assessed by evaluating dryness, crust, and keratinization. Compared to control mice, significantly lower total serum IgE levels were observed in IM mice (P < 0.01; Fig 2A, S3 Table), but there was no significant difference between the control and probiotics groups (S3 Table). Similarly, the dermatitis scores of the group control were significantly higher than that of the IM group (p < 0.01; Fig 2B, S3 Table). However, there was no significant difference between the control and probiotics groups (S3 Table).

To investigate if gut microbial richness/diversity correlates with changes in total serum IgE levels, dermatitis scores, gender, or the treatments, correlation analyses were performed. Our results indicate that the Chao estimator and PD significantly correlated with total serum IgE levels, dermatitis scores, and treatments. Moreover, the number of estimated OTUs significantly correlated with total serum IgE levels and treatments (Table 1). However, the Shannon diversity index did not correlate with any of the immunological indices and treatments evaluated. In addition, the gut microbial richness/diversity indices did not correlate with gender (Table 1). These results suggest that exposure to goat gut microbes enhances immune function, possibly by changing the richness and diversity of the mouse gut microbiota. Additionally, we observed that mouse body immunity demonstrates low correlation with the evenness of the fecal microbial community.

**Table 1:** Correlation between microbial richness/diversity and dermatitis severity indices and other variables.

	IgE	Dermatitis_Scores	treatment	Gender
(A)Chao estimator				
Spearman				
R value	-0.529**	-0.415*	0.612**	-0.006
P value	0.008	0.044	0.001	0.997
Pearson				
R value	-0.480*	-0.385	0.598**	0.070
P value	0.018	0.063	0.002	0.745
(B)Phylogenetic diversity(PD)				
Spearman				
R value	-0.521**	-0.483*	0.693**	-0.044
P value	0.009	0.017	0.000	0.840
Pearson				
R value	-0.471*	-0.471	0.680**	-0.055
P value	0.020	0.020	0.000	0.799
(C)OTU				
Spearman				
R value	-0.423*	-0.246	0.383	-0.006
P value	0.040	0.246	0.064	0.977
Pearson				
R value	-0.460*	-0.376	0.459*	0.262
P value	0.024	0.070	0.024	0.216
(D)Shannon diversity index				
Spearman				
R value	-0.122	-0.175	-0.096	-0.118
P value	0.569	0.412	0.655	0.582
Pearson				
R value	-0.198	-0.259	-0.032	-0.071
P value	0.353	0.223	0.883	0.741

P-values are from two-tailed least significant difference (*P < 0.05, **P < 0.01; n = 8).

### 3. Exposure to different microbes had limited effect on the levels of dominate gut bacteria

The individual sequences were classified based on the RDP database, and approximately 99.8% of all sequences were assigned to 23 known phyla. Firmicutes and Bacteroidetes were the two most dominate phyla. Tenericutes, Proteobacteria, Cyanobacteria and Deferribacteres phyla were also relatively abundant, as previously reported [11]. Analysis of the mean abundance levels among the experimental groups using the two-tailed least significant difference (LSD) test showed that the abundance of *Firmicutes* and *Bacteroidetes* was not significantly different between groups (S4 Table). However, the abundance levels varied substantially among the three groups for the other phyla tested. The phylum *Tenericutes* was significantly more abundant in the control group than in the IM group (p < 0.01; Panel A in S1 Fig, S4 Table), and there was no significant difference between the control and probiotics groups (S4 Table). In contrast, phylum *Proteobacteria* was more abundant in the probiotics and IM groups than in the control, and this was significant for the probiotics group (p < 0.05; Panel B in S1 Fig, S4 Table). Phylum Cyanobacteria was more abundant in the probiotics group than in the IM group (p < 0.05; Panel C in S1 Fig, S4 Table).

Among the 325 genera identified by the RDP classifier, S24-7_norank, Blautia, Ruminococcaceae_uncultured, Bacteroides, Lachnospiraceae_uncultured, Prevotellaceae_uncultured, RC9_gut_group, Oscillibacter, Ruminococcaceae_unclassified, and Alistipes were the top 10 most abundant genera in all groups (S5 Table). These comprised about 85% of total sequences (S5 Table). Among them, Oscillibacter was significantly more abundant in the probiotics group than in the control and IM groups (p < 0.05; S5 Table). Alistipes was significantly more abundant in the IM group than in the probiotics and control groups (p < 0.01; S5 Table). Among the top 11 to 20 most abundant genera, 5 genera demonstrated varied abundance between the three groups. Anaeroplasma, Defluviitaleaceae_incertae_sedis, and Erysipelotrichaceae_uncultured were significantly more abundant in the control group than in the IM group (p < 0.01, 0.05, 0.05; S5 Table). Ruminococcaceae_incertae_sedis was significantly more abundant in the probiotics group than in the IM group (p < 0.05; S5 Table). Family_XIII_incertae_sedis was significantly more abundant in the control group than in the probiotics and IM groups (p < 0.01; S5 Table).

Taken together, the results show that the dominate bacteria were largely unaffected by exposure to intestinal microbes. However, the abundance of the non-dominate species changed significantly.

### 4. Microbe exposure associated changes in gut microflora at the species level

**Fig 3:**
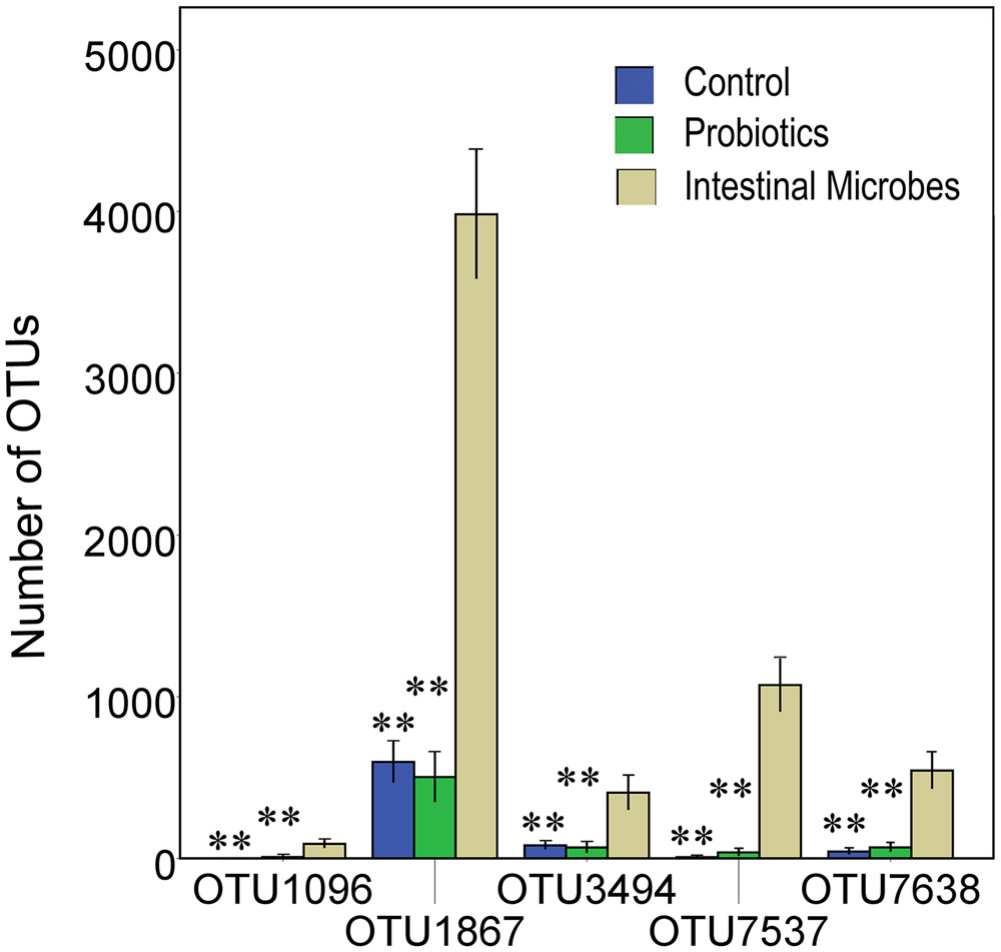
Comparison of OTU-levels in the gut microbiota between the control, probiotics, and intestinal microbes groups. (n = 8; *p < 0.05, **p < 0.01, based on a two-tailed least significant difference test).

The previous results showed minor changes in the gut microbial community at the phyla and genus levels within the three groups. The observed abundance profile of any given phylum or genus represents the sum of heterogeneous species or strains, which can lead to inaccurate interpretations. Therefore, we investigated whether we could obtain in-depth inferences based on OTU analyses. A heat map of the top 100 OTUs showed that 14 OTUs were significantly more abundant in the IM group than the other two groups (S6 Table, Fig 3). Moreover, 5 OTUs were significantly less abundant in IM samples than in the other samples (S6 Table). The OTUs mainly belong to the orders *Bacteroidales* and *Clostridiales* and genera *S24_7_norank* including 10 OTUs, *Bacteroides* including 4 OTUs, *Alistipes* 2 OTUs, *Ruminococcaceae_Incertae_Sedis* 2 OTUs, and *Ruminococcus* 1 OTU (S7 Table). All OTUs were uncultured and some still have not been assigned species names. We traced all the 19 OTUs in the sequenced bacteria of goat intestine and just found OTU6358 (*Alistipes*) appeared in the goat intest inal microbiota (S6 Table).

We also performed multivariate analyses to determine whether the changes in OTU abundance were associated with functional immunity. The results indicate that OTU1096 (S24-7_norank), OTU1867 (S24-7_norank), OTU3494 (S24-7_norank), OTU7537 (Alistipes), OTU7638 (S24-7_norank), OUT6358 (Alistipes), and OUT7378 (S24-7_norank) were significantly correlated with immunity functional indices (p < 0.05) (S8 Table). However, other OTUs significantly affected IgE concentration or dermatitis scores (p < 0.05) (S8 Table).

### 5. The gut microbial community profile is associated with exposure to goat intestinal microbes

**Fig 4:**
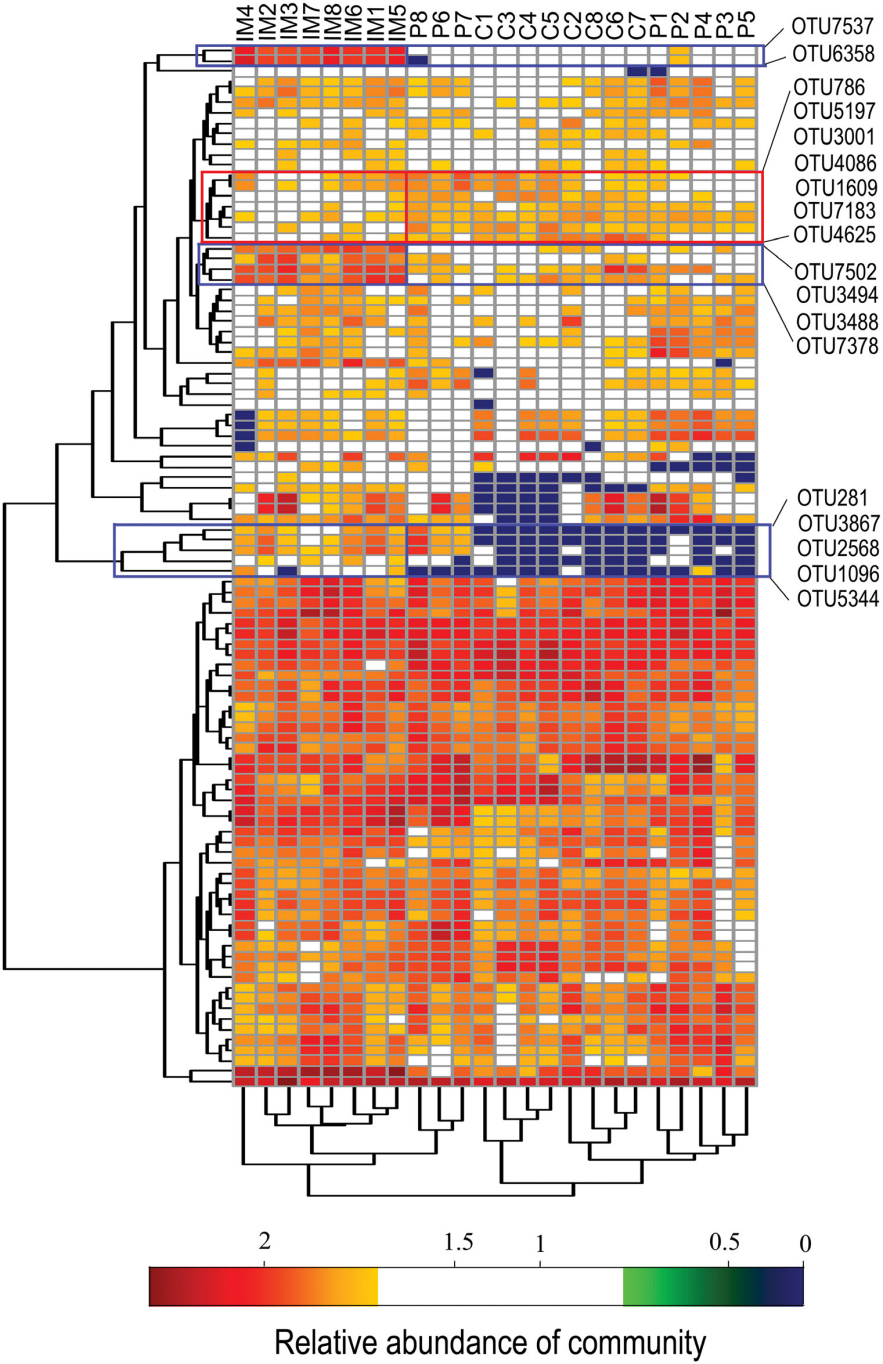
Heat map profiles and dendrograms of the most abundant OTUs in the gut microbiota for all samples. Experimental groups: control, probiotics, and intestinal microbes. Color represents relative abundance.

The intestinal microbial ecosystem is distinctively shaped by diverse microorganisms and their mutual interactions. Therefore, the related alterations in the microflora upon exposure to different microbes could be found at the community level rather than the individual microbe level. In an effort to identify the systematic differences in microbial communities between the experimental groups, the top 100 genera and all samples were hierarchically clustered based on their relative abundance (S2 Fig). We observed that all samples from the IM group were subclustered while samples from the control and probiotics groups demonstrated remarkable resemblance and could not be separated completely. For the genera, the majority of clusters showed no apparent difference between samples from the IM group and those of the control and probiotics groups. One cluster of 7 genera that included Rhizobium was less abundant in IM samples (S2 Fig, blue box), while a cluster of 2 genera, which included Roseococcus, was more abundant in these samples (S2 Fig, red box). Additionally, the top 100 OTUs and all samples were hierarchically clustered based on their abundance (Fig 4). We also observed that all samples from the IM group were subclustered, whereas samples from the other two groups could not be separated. There were three clusters, including OTU7537, OTU7502, or OTU281, which were markedly more abundant in IM samples than in other samples (Fig 4, blue box). These OTUs shared a similar pattern within each group, indicating a coherent relationship among these species or strains. Another cluster that included OTU1609 (Fig 4, red box) displayed a pattern that negatively correlated with the OTU7502 cluster (Pearson correlation coefficient r = − 0.502, Student T-test p = 0.011). These results suggest that the community-wide interrelationship of the gut microbiota is altered in the intestinal microbes of mice.

**Fig 5:**
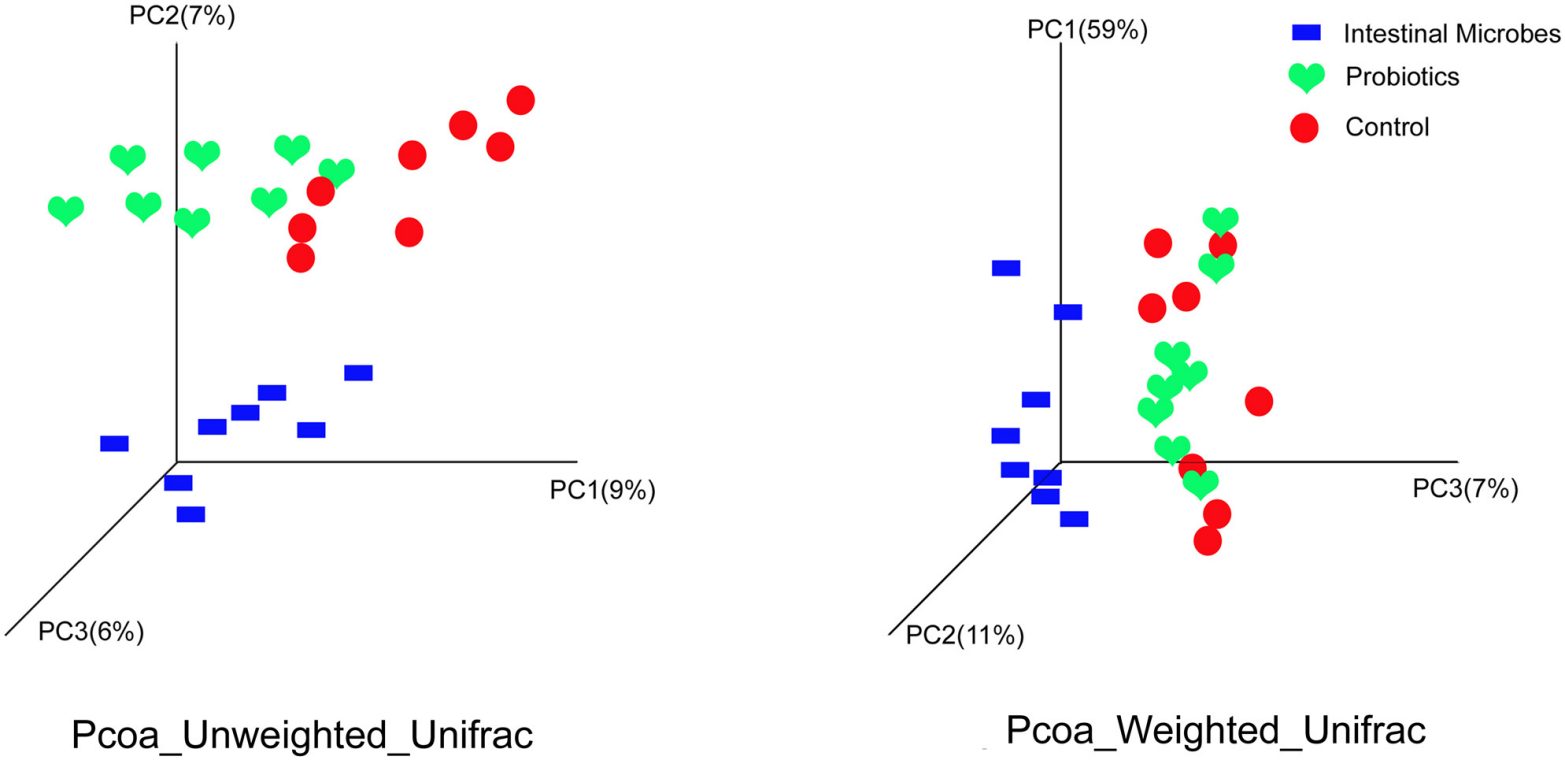
Principal coordinates analysis (PCoA) of unweighted and weighted UniFrac distances. These are for the fecal microbiota of the three experimental mice: control, probiotics, and intestinal microbes. Analysis was based on the Illumina bacterial 16S rRNA gene dataset (V4 region).

Next, we assessed differences in the total bacterial community at the single-sample level by clustering the control, probiotics, and IM samples according to their OTU abundance levels. Hierarchical cluster analysis showed that the bacterial communities were subdivided into two subclusters. The IM samples were at a close distance and some probiotics samples were clustered with control samples (S3 Fig). We also used sequence information from the 16S ribosomal RNA gene to calculate pairwise UniFrac distances between the microbial communities. We assessed both relative abundance data (weighted analysis) and the presence/absence status (unweighted analysis). Unsupervised clustering by means of principal coordinates analysis (PCoA) of UniFrac distance matrices indicated that exposure to microbes primarily explained the variation in our dataset, where the control, probiotics, and IM microbiota got separated along the principal coordinate in the unweighted analysis (Fig 5). For the weighted analysis the IM microbiota were separated from other samples and the control and probiotics were mixed. In summary, these results demonstrate that the profile of the gut microbial community was affected by microbe exposure. The results also show that probiotics had little effect on the profile of the gut microbial community.

## Discussion

In this study, we characterized the impact of exposure to goat intestinal microbes or probiotics on the diversity and composition of the gut microbiota and immune function of adult BALB/c mice, using high-throughput 16S rDNA sequencing and DNFB sensitization. We found that goat intestinal microbes have distinct effects on the richness and diversity of the gut microbiota as well as on the development of the immune system, which correlates with the microflora hypotheses. However, the three probiotics had limited effects compared with the control. Interestingly, the dominate bacteria changed slightly in IM mice, whereas the non-dominate species changed significantly, which correlated with increased immune function.

Our results suggested that microbes in the living environment influenced the structure of the gut microbial community in adult mice. According to published reports, the establishment of intestinal microbiota commences at birth, and proceeds in a sequential manner during the first years of life in humans[26]. The first bacteria to colonize the neonatal intestine are aerobic or facultative anaerobic bacteria, which initially reach high population counts. These bacteria consume oxygen, thus lowering the redox-potential in the gut, which makes way for anaerobes. Successively, a range of different anaerobic species colonizes the gut. With the establishment of an increasingly complex anaerobic microbiota, the growth of the facultatives is suppressed due to the accumulation of toxic metabolites, oxygen depletion, and substrate competition[16,26]. A number of factors influence the establishment of intestinal microbiota, including delivery and feeding modes, degree of social exposure, and environmental bacterial content[16]. The bacteria colonized in the intestine form a reservoir that seed a luminal community and dictate their composition. The delayed colonized bacteria cannot reach the high numbers that they do in the absence of competition. From our results, the living environmental microbes still affected the composition of the gut microbiota in weaned mice and were not only relevant during the first few weeks of life. It is possible that the newly colonized bacteria became members of the delayed colonization community and remained in the intestine as nondominant bacteria.

The results also showed that exposure to living environmental microbes affected whole-body immunity. According to published reports, immune cells could be activated by autochthonous flora or transient flora in the intestine[2,5], and the induction of each lymphocyte subset is regulated by a distinct component of the microbiota [5]. Greater diversity of the microbial community is associated with greater activation of immune cells, thereby increasing immune function. In our study, the microbes in the living environment may affect the immune system in two ways. First, the microbes were orally consumed by mice and became transient flora, allowing them to affect the body immune system. We believe that most exogenous goat microbes only resided in the mouse gut transiently. Second, the transient flora could directly or indirectly activate some immune effectors in the gut, such as IgA and α-defensins, which would allow some living environmental microbes to colonize in the gut as autochthonous flora. Because the composition of the microbial community in the gut could be altered by immune effectors, such as IgA and α-defensins [27], the newly colonized microbes can further increase immune function. Further studies are needed to determine whether there is a direct connection between the altered gut microbiota and resistance to DNFB sensitization.

Interestingly, our results correlate with that obtained from studies on the intestinal microbiota of autistic children compared with normal children[28] in many instances. First, autistic children had lower richness indices of OTUs, Chao estimator, and PD index, and a similar Shannon index compared with normal children[28]. Second, the abundance of dominate bacteria was not significantly different between the two groups. The cause for the close correlation between our findings and that of Kang et al.[28] requires further exploration.

After identification of different levels of OTUs between the IM and control or probiotic groups, we traced the bacteria in the sequenced intestinal microbes of goats. One OTU (*Alistipes*) was found in the 16s rRNA sequences of the goat intestinal microbiota. This result can be explained by the following observations: 1) the microbes in the goat gut only stimulated immunity in mice as transient flora, and few remained in the mouse gut; and 2) although there were many microbes in mouse gut, we only detected 200 or so, which did not represent the entire microbial community.

We also observed that there was no difference between the control and probiotics groups in the composition of the intestinal microbial community. This correlates with the findings of McNulty et al.[29]. McNulty et al. found that the bacterial species composition and some functional genes had not significantly changed in the feces of humans who consumed the commercially available fermented milk product containing four strains of probiotics before sampling [29].

In conclusion, we demonstrate that exposure to living environmental microbes is closely associated with a distinct gut microflora that can be characterized by increased richness and diversity as well as by altered composition and structure of the microbial community, especially of non-dominate bacteria. The increased richness of gut microflora is linked to higher immune function. We also show that limited probiotics had minimal effects on altering the composition of the gut microbiota.

## Supporting Information

S1 Fig Phylum-level comparison of the gut microbiota. among the Control, Probiotics and Intestinal Microbes groups.
(TIF)

S2 Fig Heat map profiles and dendrograms of top most abundant genera in gut microbiota for all samples. (TIF)

S3 Fig Dendrogram of complete-link hierarchical clustering based on the amount of OTUs. (TIF)

S1 Table Miseq data summary with the number of total sequence reads and qualified sequence reads. (XLSX)

S2 Table Microbial diversity indices with OTUs obtained by UCLUST (97% similarity). (XLSX)

S3 Table Comparison of (A) total serum IgE levels and (B) Dermatitis scores of rostral back and ear among groups Control (Con.), probiotics (Pro.), and intestinal micorbes (IM). (XLSX)

S4 Table Top 10 most abundant phyla in mice gut microbiome. (XLSX)

S5 Table Top 20 most abundant genera in mice gut microbiome. (XLSX)

S6 Table Immunity function correlated 19 OTUs in mice gut microbiome and traced in intestinal bacteria of goat. (XLSX)

S7 Table Immunity function correlated OTUs. (XLSX)

S8 Table Correlation between microbial species and immunity indexes among all samples. (XLSX)
