# Supplementary figures and images for: Impact of Environmental Microbes on the Composition of the Gut Microbiota of Adult BALB/c Mice
Source: PLoS One. 2016 Aug 12;11(8):e0160568. doi: 10.1371/journal.pone.0160568 (PMC4982623; doi:10.1371/journal.pone.0160568)

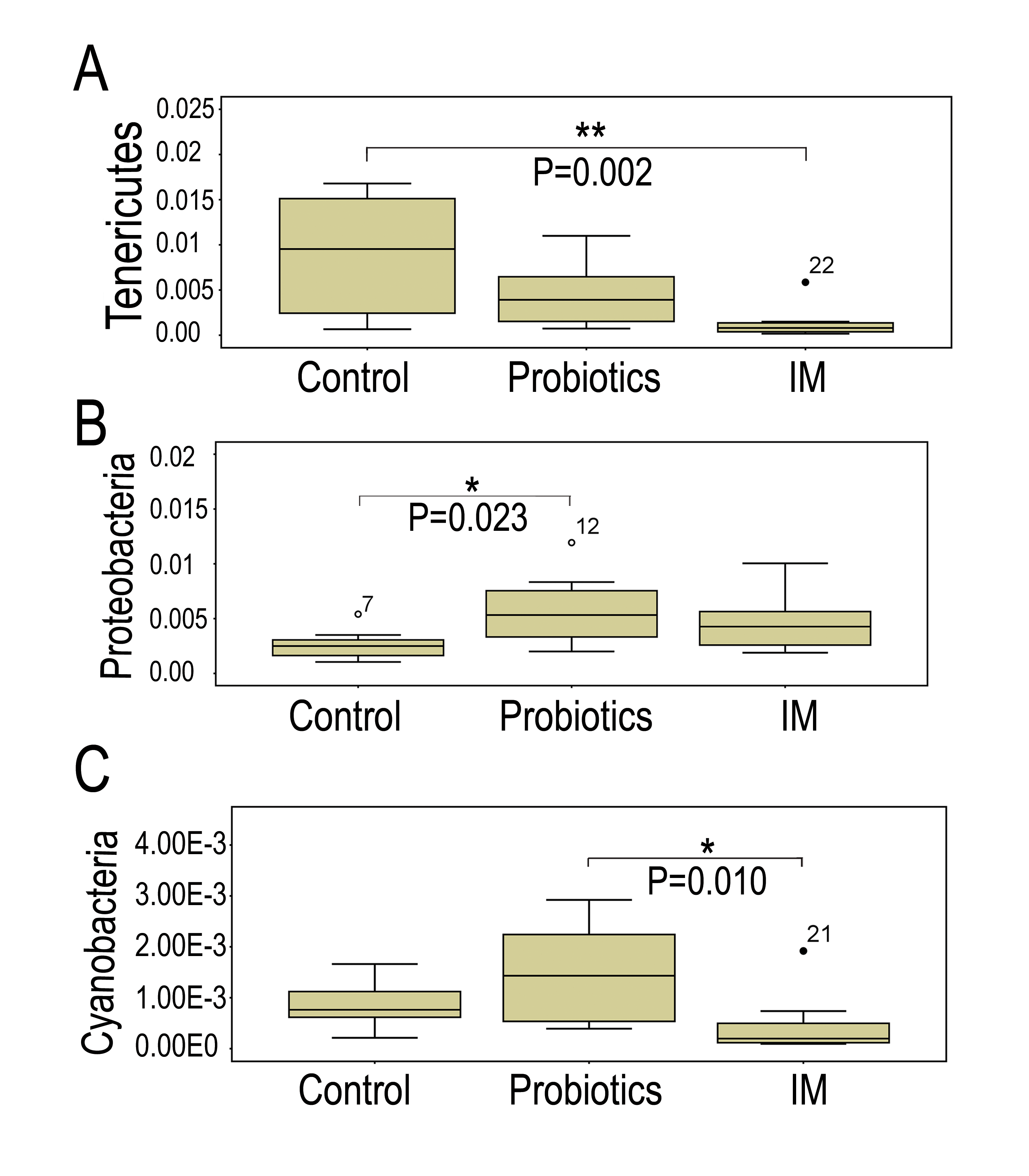

Supplement: S1 Fig — among the Control, Probiotics and Intestinal Microbes groups. (TIF) [file pone.0160568.s001.tif]

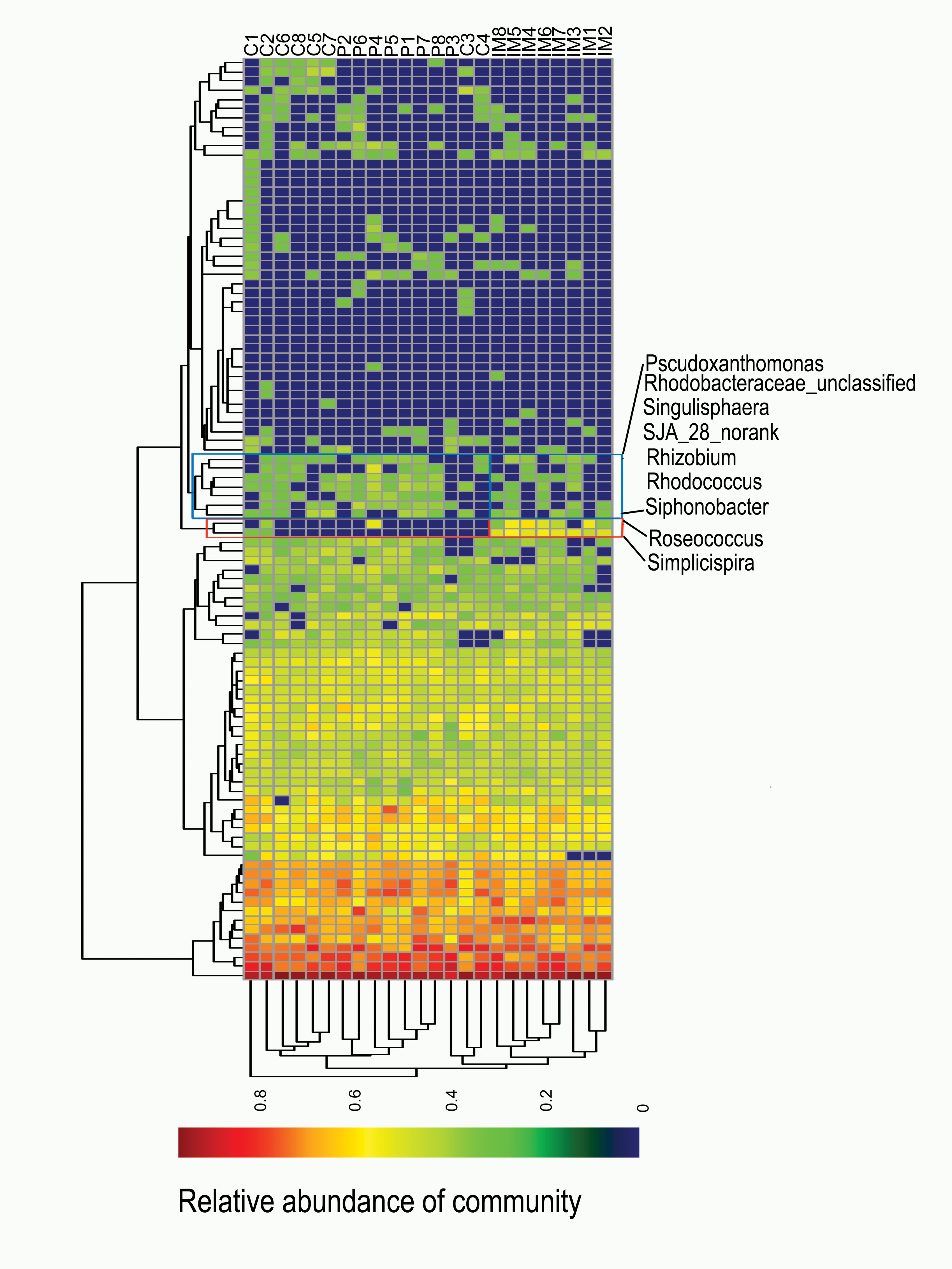

Supplement: S2 Fig — (TIF) [file pone.0160568.s002.tif]

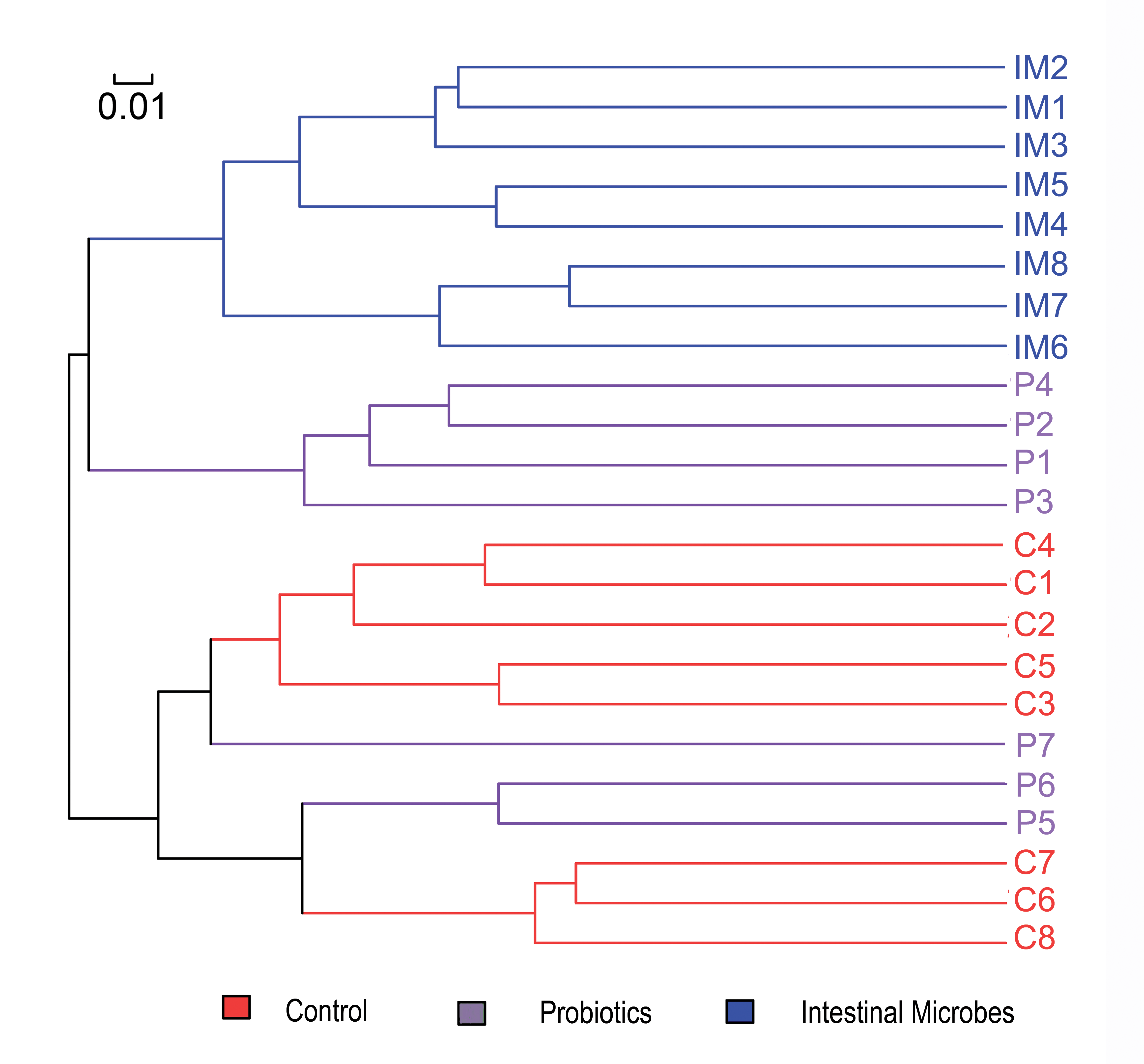

Supplement: S3 Fig — (TIF) [file pone.0160568.s003.tif]
